# Children of boom and recession and the scars to the mental health – a comparative study on the long term effects of youth unemployment

**DOI:** 10.1186/s12939-016-0305-0

**Published:** 2016-01-20

**Authors:** Pekka Virtanen, Anne Hammarström, Urban Janlert

**Affiliations:** University of Tampere, School of Health Sciences, Tampere, 33014 Finland; Department of Public Health and Clinical Medicine, Social Medicine, Umeå University, Umeå, Sweden; Department of Public Health and Clinical Medicine, Epidemiology, Umeå University, Umeå, Sweden

## Abstract

**Background:**

Earlier research shows that there is an association between unemployment and poor mental health, and that recovery from the damages to mental health obtained during unemployment remains incomplete over a long period of time. The present study relates this ‘mental health scarring’ to the trade cycle, exploring if those exposed to youth unemployment during boom differ from those exposed during recession with respect to mental health in the middle age.

**Methods:**

The sample consists of two cohorts from the same industrial town in Northern Sweden: the cohort born in 1965 and the cohort born in 1973 included all pupils attending the last grade of compulsory school, respectively, in 1981 and in 1989. Their depressiveness and anxiousness were assessed by questionnaires at age 21 and again at age 43/39. Mental health at follow-up was related to exposure to unemployment during age years 21-25. Statistical significance of the cohort*exposure interactions from binary logistic regression analyses were used to assess the cohort differences in the mental health between Cohort65 and Cohort73, entering the labour market, respectively, during a boom and a recession.

**Results:**

Compared to the unexposed, high exposure to unemployment at the age from 21 to 25 was associated to increased probability of poor mental health in the middle age in both in Cohort65 (odds ratio 2.19 [1.46-3.30] for anxiousness and 1.85 [1.25-2.74]for depressiveness) and in Cohort73 (odds ratio 2.13 [1.33-3.39] for anxiousness and 1.38 [0.89-2.14] for depressiveness). The differences between the cohorts also turned out as statistically non-significant.

**Conclusions:**

The scars of unemployment exposure onto future health seem to be rather insensitive to economic trades. Thus, at the population level this would mean that the long-term health costs that can be attributed to youth unemployment are more widespread in the generation that suffers of recession around the entry to the work life.

## Background

In his major work, “Children of the Great Depression”, Glen H Elder is describing what happened to all those children that were born in 1921 and were brought up during the great depression, 1929-39 [1]. We are given a fascinating insight of the fate of individual persons, but we could not know whether their life would have developed in another way had it not been for this severe economic crisis. Understandably, this question has been in the interest of the research utilising more recent economic fluctuations. For instance in a study among men graduated from US colleges between 1979 to 1989 [2], the consequences of leaving the school during a recession (compared to school-leavers in “normal” or “boom” period) were “large, negative and persistent”: a 1 percentage point increase in the state unemployment rate at school-leaving was estimated to lead to an annual wage loss of 2.5 % to 9 % 15 years later. A corresponding study among high school graduates [3] resulted in more moderate wage effects and replicated the finding of minimal impact on employment rates. A study focussing on the differences in mental and physical health at age 40 with reference to whether the cohort member had left the school in good or bad economy [4] found that those, in particular men, who had left school in a bad economy had worse health compared to otherwise similar counterparts who left school in times of a good economy.

The referred studies use nation and state level unemployment rate as the indicator of ‘bad economy’, or socioeconomic environment that is assumed to be associated to negative outcomes through ecological pathways, i.e. at the level of the whole cohort. Related to this, researchers interested in individual level effects of unemployment have adopted from medical vocabulary the concept ‘scarring’, referring to the adversities due to unemployment that remain - or become - ‘visible’ when the individual has passed the actual unemployment episode. In addition to repeated unemployment, the scars may appear as ‘wage penalties’ [5–7], as lost occupational and social skills and motivation [8, 9], and as downward occupational mobility [10, 11].

There are also studies that bring ‘scarring’ from the allegorical back to its original context [12–14]. The rationale of such studies is evident: the health damages obtained during unemployment cannot heal instantaneously at the end of the unemployment but the recovery takes in any case some time and may remain incomplete over a long period of time. Scarring may be assumed to depend, in addition to the amount of the exposure to unemployment, on the moment of exposure during the life course. In the concept frames of life course epidemiology [15], the years of entry into labour market may be considered as a period of particular sensitivity, during which exposure to adverse conditions may affect health later in life, independent of later circumstances. Unemployment also may harm development of young people’s identities and their socialization into the adult world, which may program them psychologically for latent outcomes that appear with delay [16, 17]. Moreover, there may be ‘ecological modification’ [18] of the effects: as the impact of unemployment evidently depends on unemployment rate in the community where individuals live [19], it is possible that corresponding difference can be seen in the scarring.

Our starting point in the present study is that national macroeconomic status constitutes the ecological context. We link fluctuations in the macroeconomy to the life-course of the individuals and explore if the ‘mental health scars’ of those exposed to youth unemployment during a boom differ from the scars due to the exposure during a recession. More specifically, we are asking if there is difference in scarring of the mental health due to exposure to unemployment during the age years 21-25 between those exposed during the boom in 1986-1990, and those exposed during the recession in 1994-1998. Our hypothesis is that exposure during boom is associated to worse scarring than exposure during recession.

## Method

### Sample and data collection

The sample consists of two cohorts including all pupils aged 16 in the last year of compulsory school (grade nine) from the same middle-sized industrial town of Northern Sweden: cohort65 (born in 1965) finished grade nine in 1981 to cohort73 (born in 1973) in 1989. The cohorts were investigated with extensive questionnaires at age 21 and in adult age (age 43 in Cohort65, age 39 in Cohort73). The response rate at age 21 was 97.9 % in Cohort65 and 90.0 % in Cohort73. The response rates (of those still alive) in adult age were, respectively, 94.3 % (n = 1001) and 85.6 % (n = 686). Cohort65 was also investigated with extensive questionnaires at other ages, e.g. at age 30 and at age 42. In addition to the survey data, register data about the number of days in unemployment per year were obtained from the Longitudinal Integration Database for Sick Leave and Labour Market Studies (LISA) from Statistics Sweden for each cohort participant living in Sweden. This register data was available from 1992 and onwards.

The study has been approved by the Regional Ethical Review Board in Umeå, Sweden.

### Exposure to unemployment

**Fig. 1:**
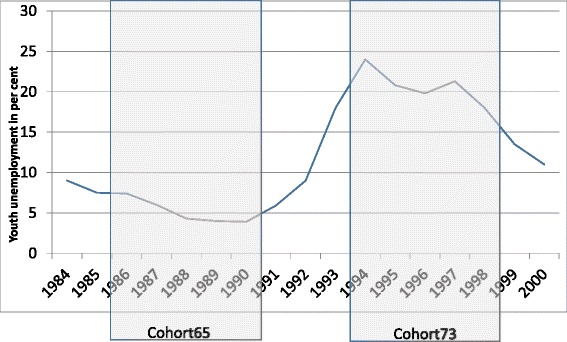
Nation level rate of youth unemployment from 1984 to 2000 in relation to the exposure window from age 21 to age 25 of Cohort65 and Cohort73

Exposure to unemployment was measured during the age window from 21 to 25 years, representing calendar years 1986-1990 for Cohort65 and 1994-1998 for Cohort73. The cohorts reached age of 21 at very different phases of the trade cycle, as demonstrated by national figures of youth unemployment (Fig. 1). For Cohort65, in 1986, the figure was about 7 per cent, and for Cohort73, in 1994, over 20 per cent. Four years later, at age 25, the rates of youth unemployment were about 4 per cent and 14 per cent, respectively.

Cohort65 was asked in the survey in 1995 with a matrix, including the options student, at work, unemployed and other, in which situation(s) they had been during the first and the second half of each year since the previous survey in 1986 [20]. If unemployment was the only option, it was considered to last the whole half-year period; if there were other option(s), unemployment was assumed to last corresponding proportion of the period. A total of 730 (73 %) respondents had not experienced unemployment. The rest were classified at the median as having ‘low exposure’ (8-24 weeks, n = 128) or ‘high exposure’ (25-183 weeks, n = 143) to unemployment between age 21 and age 25 (1986 and 1990).

For Cohort73, register data was available to measure the exposure to unemployment. The total time they had received unemployment allowance during the calendar years from 1994 to 1998 was classified, as for Cohort65, into ‘no’ (n = 266, 39 %), ‘low’ (1-15 weeks, n = 209) and ‘high’ (16-126 weeks, n = 211) exposure.

### Indicators of mental health

The questions about symptoms of mental health [21, 22] used at baseline were kept unchanged at the follow-up surveys. Two composite measures of internalized symptoms at age 16, distinguishing depressiveness and anxiousness, were created on basis of the symptom clusters defined in DSM-5 [23] (see [24]). The items concerning (i) ‘sleeplessness’, (ii) ‘poor appetite’, (iii) ‘fatigue’, (iv) ’concentration difficulties’, (v) ‘felt down or sad’ and (vi) ‘feeling downhearted about future’ were calculated into the depressiveness score, and the items concerning (i) ’restlessness’, (ii) ’concentration difficulties’, (iii) ’worry’, (iv) ‘palpitations‘ and (v) ’panic’ into the anxiousness score. After pooling the cohorts, those belonging to the upper quartiles were defined as suffering from depressiveness/anxiousness.

### Statistics

Associations between exposure to unemployment in the youth and depressiveness and anxiousness in the middle age were examined by binary logistic regression analyses. P-values for the cohort*exposure interactions were used to assess the between-cohort differences in the associations. The analyses were adjusted for gender, parental socioeconomic status (no, one or both having a blue collar occupation), respective mental health at baseline and exposure to unemployment during the three years preceding the follow-up.

## Results

The cohorts are described in Table 1. Cohort73 members originated more often from a family of one blue collar parent and less often from a family of no blue-collar parents, and as expected, exposure to youth unemployment was more common among them than in Cohort65. At the follow-up, unemployment during the three preceding years was very rare in Cohort73, whereas in Cohort65 one of ten had experienced unemployment. As regards mental health, at the baseline both depressiveness and anxiousness were more common in Cohort73; at the follow-up there was an opposite difference in depressiveness and no difference in anxiousness.

**Table 1:** Descriptive statistics of the cohorts

	Cohort65 (n = 1001)	Cohort73 (n = 686)
Gender		
- men	52 %	50 %
- women	48 %	50 %
Parents’ occupational status		
- both blue-collar workers	37	38
- one blue-collar worker	33	39
- not blue-collar workers	30	24
Poor mental health at baseline (1)		
- anxiousness	18 %	25 %
- depressiveness	23 %	34 %
Unemployment at baseline		
- exposed	27 %	61 %
- median among the exposed	25 weeks	16 weeks
Poor mental health at follow-up (1)		
- anxiousness	24 %	23 %
- depressiveness	33 %	25 %
Unemployment at follow-up(2)		
- exposed	10 %	1 %
- median among the exposed	50 weeks	20 weeks

(1) Score in the upper quartile

(2) During the three years that precede the follow-up

The regression analyses for anxiousness revealed that in both groups high exposure to youth unemployment was associated to upper quartile score in the middle age (Table 2). The odds ratios remained almost unchanged when adjusted for gender and parents’ socioeconomic status, and were reduced but still statistically significant when anxiousness at the baseline and recent unemployment at the follow-up were added to the model. Patterns of the associations were quite similar, and also the p-value of the group*exposure interaction from the fully adjusted model (p = 0.729) showed the difference between the groups as non-significant.

**Table 2:** Anxiousness (score in the upper quartile) in the forties according to exposure to unemployment at age 21-25 during the boom in 1986-1990 (Cohort65) and during the recession in 1994-1998 (Cohort73)

Exposure	Cohort65				Cohort73			
	Anxi	OR (1)	OR (2)	OR (3)	Anxi	OR (1)	OR (2)	OR (3)
Zero	21 %	1	1	1	17 %	1	1	1
Low	28 %	1.46 (0.95-2.24)	1.45 (0.94-2.24)	1.28 (0.82-1.99)	24 %	1.59 (1.01-2.50)	1.59 (1.01-2.51)	1.63 (1.01-2.62)
High	41 %	2.65 (1.81-3.88)	2.65 (1.80-3.92)	2.19 (1.46-3.30)	31 %	2.25 (1.45-3.47)	2.33 (1.50-3.63)	2.13 (1.33-3.39)

(1) Unadjusted

(2) Adjusted for sex

(3) adjusted for (2) + baseline anxiousness + recent (last three year’s) unemployment at follow-up

In corresponding analyses for depressiveness, high exposure to unemployment in the youth predicted depressiveness in the middle age in Cohort65 but not in Cohort73 (Table 3). The difference between the groups was, however, non-significant (p-value of the group*exposure interaction 0.524).

**Table 3:** Depressiveness (score in the upper quartile) in the forties according to exposure to unemployment at age 21-25 during the boom in 1986-1990 (Cohort65) and during the recession in 1994-1998 (Cohort73)

Exposure	Cohort65				Cohort73			
	Depr	OR (1)	OR (2)	OR (3)	Depr	OR (1)	OR (2)	OR (3)
Zero	29 %	1	1	1	21 %	1	1	1
Low	39 %	1.52 (1.02-2.25)	1.51 (1.02-2.25)	1.43 (0.95-2.15)	25 %	1.24 (0.79-1.89)	1.24 (0.80-1.90)	1.14 (0.73-1.78)
High	49 %	2.43 (1.68-3.50)	2.37 (1.63-3.45)	1.85 (1.25-2.74)	28 %	1.46 (0.96-2.21)	1.55 (1.01-2.37)	1.38 (0.89-2.14)

(1) unadjusted

(2) adjusted for sex and parents occupational status

(3) adjusted for (2) + baseline depressiveness + recent (last three years’) unemployment at follow-up trade cycle*exposure interaction, p = 0.414

## Discussion

This study compared population cohorts entering the labour market during a boom and during a recession with respect to mental health in the middle age. We found that the mental health scars left by exposure to unemployment at the age from 21 to 25 years remained irrespective of the trade cycle during those years.

In terms of theory, design of the study was based on two conceptions. First, youth was assumed to be an important phase of the life-course as regards individual’s development into a ‘labour market citizen’, and disturbances in this development were assumed as particularly stressful. Second, this stress could be assumed to depend on ecology of the unemployment, being particularly strong during boom, because then the unemployment is experienced as an individual failure and stigma rather than as a collectively shared misfortune, [19, 25]. The results are in line with the scarring hypothesis and earlier findings with Cohort65 [13, 26]. We also found that the scarring depends on amount of the exposure, being not significant among those experiencing relatively short-term unemployment. But we did not get support for the hypothesis that those exposed during recession would recover rather soon, whereas the scars from exposure during boom would turn out as longer-lasting. This zero finding goes well into the results of Novo et al. [27] which show that that exposure to long-term unemployment since age 16 is associated to poor health at age 21 equally in the boom and Cohort73.

The study concentrated on mental health, with two scores of the internalised symptoms as the outcomes. Non-significant between-cohort difference with both depressiveness and anxiousness supports the conclusion that the scarring is independent of the trade cycles. Moreover, when corresponding analyses were performed with cut-off points at 50 % and at 90 %, the cohort*exposure interactions were as well non-significant.

Health selection into and out of unemployment has been demonstrated in Cohort65 [28], and is evident also in Cohort73. In order to control such ‘reverse scarring’, or poor mental health as the cause of unemployment, we adjusted the analyses for respective mental health variable at the baseline of the exposure window. This reduced, as expected, the odds ratios in both groups. Moreover, in order to control the confounding due to exposure in the adulthood, the analyses were adjusted for unemployment during the time window of three years prior to the follow-up. Also duration of the follow-up was time was, although not exactly the same (22 years for Cohort65 and 18 years for Cohort73), long enough to study scarring; we also believe that four years age difference (43 vs 39) was of minor importance for our findings. A study from US indicates that women, more easily than men, can adjust the balance between work and family, in particular during recession [3]. This may protect them against mental health scarring. On the other hand, in the Swedish context men and women seem to be quite equally hit by the health consequences of unemployment [29]. Therefore, men and women were not studied separately, but gender was treated as confounder in the analyses. Swedish context, may also explain why we could not replicate the finding of a corresponding analysis from US [4]. The ‘Scandinavian’ social security, with relatively generous benefits, may have mitigated the effects of unemployment in both cohorts. On the other hand, as the response to the recession, the active labour market policy measures were intensified. Our result indicates, however, that this had not prevented mental health scarring. An important way to protect people from the effects of open unemployment is increased enrolment into post-basic schooling [30]. In Sweden the post-basic education posts were also increased as a response to the recession, as is evident also in the studied groups: 48 % of Cohort73 had completed secondary high (3-4 years) education at the baseline in 1995 whereas corresponding figure in Cohort65 in 1986 was 31 %. In fact, only 33 % of the Cohort73 but 60 % of Cohort65 were employed at the baseline; therefore we could not define their socioeconomic status, and used parents’ socioeconomic status as a proxy.

Unemployed and otherwise underprivileged people tend to be overrepresented among the non-responders in survey studies. This is why the low attrition rate is an especially important strength of this study. The very high response rates of Cohort65 were no more reached in the surveys of Cohort73. Due to this, it is probable that more of the individuals with poor mental health were included in Cohort65 than in Cohort73; however, we can assume that there was such difference both at baseline and at follow-up, and consequently can assume that adjustment for baseline mental health removed the effect of the difference. Nevertheless, dropout due to depressiveness or anxiousness in Cohort73 may have biased positively their mental health by the follow-up survey. This may partly explain why their mental health is relatively good, irrespective of the exposure. It is, however, unlikely that the results would be an artefact, given the clearly significant findings about scarring in both groups and the clearly non-significant between-group difference.

For Cohort65, exposure to unemployment was based on survey data, as no register data about their unemployment in 1986-1990 was available. Also it is not relevant to compare the cohorts in terms of absolute amount of exposure. However, it is possible to use the variables as a basis for classification into those with no, low and high exposure: it is likely that the unexposed in both cohorts become classified accurately, and also dichotomization of the exposed at median can be considered to classify them quite correctly into groups with high and low exposure.

Cohort65 had experienced more unemployment during the years preceding the follow-up. The confounding effect of this differential unemployment exposure on mental health of the cohorts was controlled by adding it in the statistical model. As to the reasons of the difference, one obvious explanation is clearly higher education of cohort73. Moreover, it is possible that the unemployed tended to drop out from cohort73, while in cohort65 corresponding dropout vas not possible by virtue of the high response rates.

Some studies call into question whether leaving school in a bad economy will hurt health. Papers by Ruhm and others (e.g. [31, 32]) show that physical health and health behaviours improve as the unemployment rate rises in society. However, these findings have been questioned, as the “positive” findings of unemployment in most cases are based on ecological studies, while “negative” findings are based on individual studies [33].

There are, to our knowledge, no earlier studies that have combined in similar way the individual and the ecological perspectives in research about the health effects of unemployment. The Northern Swedish Cohort has been shown to be representative to Sweden’s population [34], and the results of this study can be generalized to countries with similar labour market structures. Nevertheless, the results raise need to replication studies in different historical and national contexts.

## Conclusion

The scars of unemployment exposure onto future health seem to be rather insensitive to economic trades. Thus, at the population level this would mean that the long-term health costs that can be attributed to youth unemployment are more widespread in the generation that suffers of recession around the entry to the work life.
